# Galangin suppresses RANKL‐induced osteoclastogenesis via inhibiting MAPK and NF‐κB signalling pathways

**DOI:** 10.1111/jcmm.16430

**Published:** 2021-05-03

**Authors:** Xiucheng Li, Jiawei Jiang, Zhifan Yang, Songtao Jin, Xuanyuan Lu, Yu Qian

**Affiliations:** ^1^ Department of Orthopaedics Shaoxing People's Hospital Zhejiang University School of Medicine Shaoxing China

**Keywords:** galangin, MAPK, NF‐κB, osteoclast, osteoclastogenesis

## Abstract

Osteoclasts play a critical role in osteoporosis; thus, inhibiting osteoclastogenesis is a therapeutic strategy for osteoporosis. Galangin, a natural bioflavonoid extracted from a traditional Chinese herb, possesses a variety of biological activities, including anti‐inflammation and anti‐oxidation. However, its effects on osteoporosis have not been elucidated. In this study, we found that galangin treatment dose‐dependently decreased osteoclastogenesis in bone marrow–derived macrophages (BMMs). Moreover, during osteoclastogenesis, osteoclast‐specific genes, such as tartrate‐resistant acid phosphatase (TRAP), cathepsin K (CtsK), ATPase, H + transporting, lysosomal V0 subunit D2 (V‐ATPase d2) and dendritic cell–specific transmembrane protein (DC‐STAMP), were down‐regulated by galangin treatment. Furthermore, the results of the pit formation assay and F‐actin ring staining revealed impaired osteoclastic bone resorption in the galangin‐treated group compared with that in the control group. Additionally, galangin treatment also inhibited the phosphorylation of p38 and ERK of MAPK signalling pathway, as well as downstream factors of NFATc1, C‐Jun and C‐Fos. Consistent with our in vitro results, galangin suppressed lipopolysaccharide (LPS)‐induced bone resorption via inhibition of osteoclastogenesis. Taken together, our findings provide evidence that galangin is a promising natural compound for the treatment of osteoporosis and may be associated with the inhibition of MAPK and NF‐κB signalling pathways.

## 1 INTRODUCTION

Osteoporosis is a group of diseases in which the bone mass is significantly reduced, predisposing patients to spontaneous bone fragility and bone fractures. It has been reported that more than 70 million people worldwide are affected by this life‐threatening problem.^1^ The integrity and structure of the skeleton are precisely regulated by osteoclastogenesis (mediated by osteoclasts) coupled with osteogenesis (mediated by osteoblasts).^2^, ^3^ However, excessive bone resorption by osteoclasts destabilizes this balance, ultimately causing chronic lytic diseases such as osteoporosis.^4^ Therefore, identification of agents that can modulate the formation and activity of osteoclasts is important for the treatment of osteoporosis. Osteoclasts, if not exclusive, are the primary bone‐resorbing cells. During the process of osteoclastogenesis, osteoclasts degrade the bone matrix by producing and secreting cathepsin K (CtsK) and tartrate‐resistant acid phosphatase (TRAP), making new space for osteogenesis, and stimulate osteoblast differentiation.^5^ In the majority of osteoporosis cases, osteoclasts are extensively activated, resulting in increased bone resorption, leading to bone mass loss when bone resorption activities exceed bone formation activities. Currently, oestrogen‐replacement therapy and bisphosphonates are widely used in the clinic because of their effective inhibition of osteoclastogenesis and prevention of bone mass loss.^6^ However, it has been reported that oestrogen‐replacement therapy increases the risk of post‐menopausal breast cancer, which makes such therapy a difficult choice for both patients and doctors.^7^ The adverse effects of bisphosphonates present another challenge for the management of osteoporosis.^8^, ^9^ Osteonecrosis of the jaw is a serious side‐effect related to the use of bisphosphonates, exposing patients to pain, soft tissue swelling, bone exposure and infection. Once osteonecrosis is established, treatment is difficult and is without well‐established protocols that assure absolute therapeutic success.^10^ Thus, searching for a safer and more effective therapeutic target is urgently needed.

**FIGURE 1:**
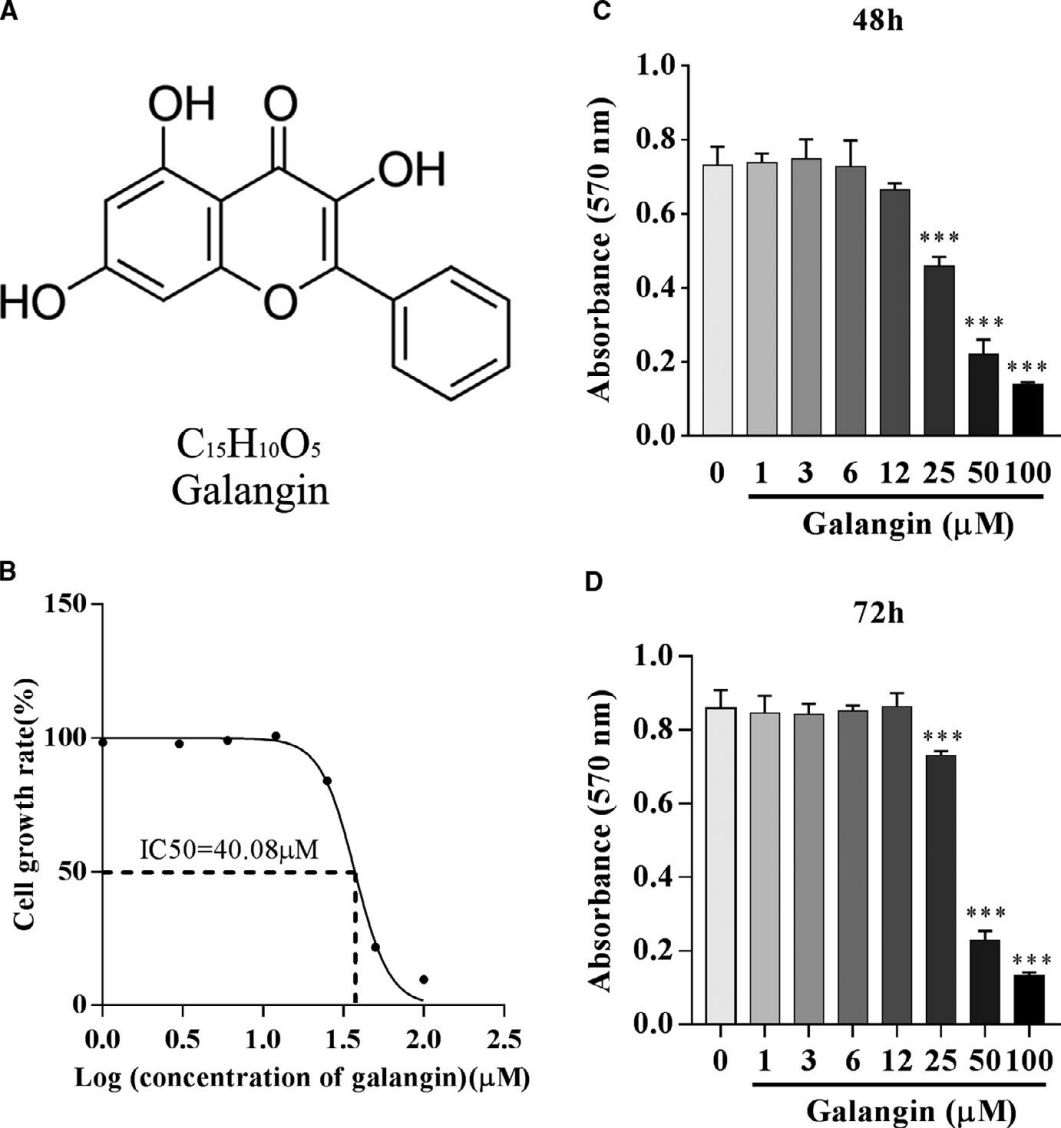
Effect of galangin on the viability of BMMs. (A) The molecular structure of galangin. (B) IC_50_ values obtained for the activity of galangin against BMMs. (C‐D) MTT assays were performed to determine the cytotoxicity of galangin on BMMs after treatment for 48 h and 72 h. Data are represented as the mean ± SD ***P < .001, compared with control group

Galangin (3,5,7‐trihydroxyflavone, Figure 1A) is a natural bioflavonoid that is primarily extracted from the rhizomes of *Alpinia officinarum*, a herbal medicine that has been used in Asia for decades.^11^ Previous studies have shown that the bioflavonoid compounds quercitrin and taxifolin inhibit osteoclasts.^12^ Belonging to the family of bioflavonoids, galangin possesses antibacterial,^13^ anti‐inflammatory^14^ and antiviral^15^ activities, and inhibits a variety of tumour cells.^16^, ^17^, ^18^ Galangin inhibits collagen‐induced arthritis and prevents osteoclastic bone resorption through enhancement of osteoblast‐induced TNF receptor superfamily member 11b (OPG) expression.^19^ Thus, we were interested in whether galangin inhibits the functions of osteoclasts. The aim of this study was to evaluate the effects of galangin on the differentiation and bone resorption activity of osteoclasts, and to explore its underlying mechanisms in vivo and in vitro.

## 2 MATERIALS AND METHODS

### 2.1 Cell culture

Primary mouse bone marrow macrophages (BMMs) were isolated from the femurs and tibiae of C57BL/6 mice as previously described.^20^ BMMs were maintained in alpha‐modified minimal essential medium (α‐MEM) supplemented with 1% penicillin‐streptomycin, 10% foetal bovine serum (FBS) (Gibco) and 30 ng/mL monocyte‐macrophage colony‐stimulating factor (M‐CSF) (PeproTech). Cells were seeded into 96‐well plates at a density of 8 × 10^3^/well, or 6‐well plates at a density of 3 × 10^5^/well for the following procedures.

### 2.2 Cell viability

Cell viability was evaluated using a 3‐(4,5‐dimethylthiazol‐2‐yl)‐2,5‐diphenyl tetrazolium bromide (MTT) assay. Cells were seeded into 96‐well plates and treated with 0, 1, 3, 6, 12, 25, 50 or 100 μmol/L galangin (Sigma‐Aldrich St. Louis, MO, USA) for 48 hours and 72 hours. At each established time‐point, the culture medium was replaced by dimethyl sulfoxide solution to solubilize crystals (DMSO). Then, the absorbance was measured at a wavelength of 570 nm.

### 2.3 TRAP staining

Cells were seeded into 96‐well plates, treated with 0, 3, 6 or 12 μmol/L galangin, and supplemented with 30 ng/mL M‐CSF and 100 ng/mL RANKL (PeproTech, Rocky Hill, NJ, USA). The medium was replaced every other day for six days. The cells were fixed with 4% paraformaldehyde for 10 minutes, washed with phosphate‐buffered saline (PBS) twice and stained for TRAP. The images of TRAP‐positive multinucleated cells were counted, and the surface areas of osteoclasts per field were calculated using Image‐Pro Plus software (Media Cybernetics).

### 2.4 Actin ring staining

An F‐actin staining kit (Millipore, Darmstadt, Germany) was used according to the manufacturer's protocol. BMMs were treated with 0, 3, 6 or 12 μmol/L galangin, supplemented with 30 ng/mL M‐CSF and 100 ng/mL RANKL for six days. Then, cells were fixed with 4% paraformaldehyde for 10 minutes and permeabilized with 0.1% Triton X‐100 for 5 minutes. After rinsing with PBS three times, cells were treated with 100 μL iFluor 488‐Phalloidin working solution for 40 minutes. Cells were rinsed with PBS three times, and then 6‐diamidino‐2‐phenylindole (DAPI) staining solution was added for 5 minutes. Images were acquired under a fluorescence microscope (Eclipse TS100; Nikon).

### 2.5 Bone resorption assays

Cells were seeded into 96‐well plates containing 100‐μm bone slices (Rongzhi Haida Biotech Co., Ltd), treated with 0, 3, 6 or 12 μmol/L galangin and supplemented with 30 ng/mL M‐CSF and 100 ng/mLf RANKL. Cells were removed by mechanical agitation and sonication until matured osteoclasts were formed. Bone resorption pits were observed under a scanning electron microscope (SEM; Field Environmental Instruments (FEI) Inc, Hillsboro, OR, USA) and the areas of pits were measured by Image‐Pro Plus software.

### 2.6 Immunofluorescence assay

BMMs were seeded (1 × 10^4^ cells/well) in a 24‐well plate and incubated in a basal medium for 24 hours. After pre‐treatment with 12 μmol/L galangin for 30 mins, 100 ng/mL RANKL was added and the wells were cultured for 30 mins. Then, the cells were fixed with 4% PFA for 30 mins, rinsed with PBS, and 5% BSA was used for 2h at room temperature to block non‐specific binding sites. Then, cells were incubated with anti‐p65 antibody at 4℃ overnight. After using PBS washing three times, the cells were incubated for 2 hours at room temperature with secondary antibodies and stained with DAPI for 5 mins in the dark. Pictures of p65 nuclear translocation were obtained using an immunofluorescence microscope.

### 2.7 RNA extraction and qRT‐PCR experiments

Total RNA was extracted from osteoclasts using RNAiso Plus Reagent (Life Technologies, Carlsbad, CA, USA) according to the manufacturer's protocol. RNA was reverse‐transcribed to cDNA using a PrimeScript RT Reagent Kit (Takara, Shiga, Japan). Cycling parameters were 94°C, 20 seconds; 60°C, 20 seconds; and 72°C, 30 seconds for 40 cycles. The results were normalized to β‐actin, an internal housekeeping gene. The specific primers used in this study were as follows: TRAP (forward: 5'‐GCAACATCCCCTGGTATGTG‐3'; reverse: 5'‐GCAAACGGTAGTAAGGGCTG‐3'), CtsK (forward: 5'‐CTTCCAATACGTGCAGCAGA‐3'; reverse: 5'‐TCTTCAGGGCTTTCTCGTTC‐3'), V‐ATPase d2 (forward: 5'‐GAAGCTGTCAACATTGCAGA‐3'; reverse: 5'‐TCACCGTGATCCTTGCAGAAT‐3'), DC‐STAMP (forward: 5'‐AAAACCCTTGGGCTGTTCTT‐3'; reverse: 5'‐AATCATGGACGACTCCTTGG‐3') and β‐actin (forward: 5'‐GATCTGGCACCACACCTTCT‐3'; reverse: 5'‐GGGGTGTTGAAGGTCTCAAA‐3'). The qRT‐PCR results were analysed using ΔΔCT method.

### 2.8 Western blot analysis

The cells were pre‐treated with 12 µmol/L galangin for 1 hours. Untreated cells were used as a control. Then, BMMs were stimulated with 30 ng/mL M‐CSF and 100 ng/ml RANKL for 0, 5, 10, 20, 30 or 60 minutes, respectively. To investigate the dose‐dependent effects of galangin, BMMs were pre‐treated with different concentrations (0, 3, 6, 12 μmol/L) of galangin for 1 hours and then stimulated with RANKL for 30 minutes. To determine the effect of galangin on NFTAc1, C‐Jun and C‐Fos, BMMs were treated with 100 ng/mL RANKL with or without 6 µmol/L galangin for 3 days. Total cellular proteins were extracted from cultured cells using RIPA lysis buffer. Lysates were centrifuged at 12 000 g for 10 minutes at 4°C, and the supernatants were collected and mixed with SDS‐sampling buffer, followed by incubation at 100°C for 5 minutes. Samples were then resolved by SDS‐PAGE gels and transferred into nitrocellulose membranes via electroblotting. Membranes were blocked with 5% skim milk for 2 hours and probed with primary antibodies overnight at 4°C. Membranes were then washed and incubated with HRP‐conjugated secondary antibodies for 2 hours. Immunoreactivity detection was performed using a LAS‐4000 Science Imaging System (Fujifilm, Tokyo, Japan), and the obtained images were analysed with ImageJ.

### 2.9 LPS‐induced calvarial osteolysis mice model

An lipopolysaccharide (LPS)‐induced calvarial osteolysis mouse model was established,^21^ as described, to determine the inhibitory effects of galangin on osteolysis. All animal experimental procedures were performed in accordance with the principles of the National Institutes of Health (NIH) Guide for the Care and Use of Laboratory Animals and the Shaoxing Hospital of Zhejiang University (Shaoxing, Zhejiang) guidelines for animal treatments. The protocol was approved by the Zhejiang University Institutional Animal Care and Use Committee (No. 11 897). Twenty‐four 8‐week‐old C57/BL6 mice were purchased from the Shanghai SLAC Laboratory (Shanghai, China) and maintained in a controlled environment (22‐24℃, 50‐60% humidity with a 12‐h light/dark cycle) and supplied with clean food and water. Firstly, galangin was dissolved in DMSO and then diluted in PBS. Each group was received subcutaneous injection with 200 μL volume. Briefly, C57BL/J6 mice were randomly divided into four groups: sham (injection with PBS only), LPS (treatment with 5 mg/kg LPS), low‐dose galangin (treatment with 5 mg/kg LPS and injection of 2.5 mg/kg galangin) and high‐dose galangin (treatment with 5 mg/kg LPS and injection of 10 mg/kg galangin). The mice received subcutaneous injections over the sagittal midline suture of the calvarium under light anaesthesia. PBS and galangin were injected 1 day before the injection of LPS (prophylactic treatment), and subsequently, every other day over a seven‐day period. At day seven, all mice were killed and the calvaria were separated and fixed with 4% PFA for micro‐computed tomography (CT) and histological analysis.

### 2.10 Micro‐CT scanning

The calvaria was separated and fixed with 4% paraformaldehyde for three days. The fixed calvaria samples were analysed using a high‐resolution μCT scanner (Scanco Microct u100, Switzerland). Image acquisition was conducted at 70 kV and 200 μA with an isometric resolution of 20 μm. After reconstruction, a square ROI (region of interest) was selected for further analysis. The percentage of bone volume/tissue volume (BV/TV %), the number of porosities and percentage of total porosity of each sample were measured as reported previously.

### 2.11 Histomorphometry analysis

The samples were decalcified in 10% EDTA for two weeks and embedded in paraffin. Histological sections were prepared for TRAP and HE staining. The sections were photographed, histomorphometric analyses of BV/TV (%) were performed, and the number of TRAP‐position OCs and OC per bone surface (OC/BS) were determined utilizing ImageJ software.

### 2.12 Statistical analysis

All data are presented as the means ± SD The differences between two groups were evaluated by unpaired, two‐tailed Student's *t* tests, and one‐way analysis of variance (ANOVA) with LSD tests were used for multiple comparisons, with *P* < .05 considered to be statistically significant. All experiments were repeated at least three times. Statistical analysis was performed using SPSS software version 19.0.

## 3 RESULTS

### 3.1 Cytotoxic effects of galangin on bone marrow–derived macrophages

The chemical formula of galangin is shown in Figure 1A. The MTT assay was performed to analyse the potential cytotoxicity of galangin against BMMs. As shown in Figure 1B, the half‐maximal inhibitory concentration (IC50) of galangin was calculated to be 44.08 μmol/L after treatment for 72 hours. Meanwhile, the OD570nm at the 72 hours time‐point remained at 0.86 ± 0.04 (*P* = .998) in the 12 μmol/L galangin‐treated group, and it remained stable in the 6, 3 and 1 μmol/L galangin‐treated groups relative to the control group (0.86 ± 0.05) (Figure 1D). The OD_570nm_ at 72 hours dropped to 0.73 ± 0.01 (*P* < .001), 0.23 ± 0.02 (*P* < .001) and 0.13 ± 0.01 (*P* < .001) in the 25, 50 and 100 μmol/L galangin‐treated groups, respectively. A similar trend was observed at the 48 hours time‐point (Figure 1C). These results indicate that the viability of BMMs is not affected by galangin at concentrations up to 12 μmol/L.

### 3.2 Galangin inhibits RANKL‐induced osteoclast formation in vitro

**FIGURE 2:**
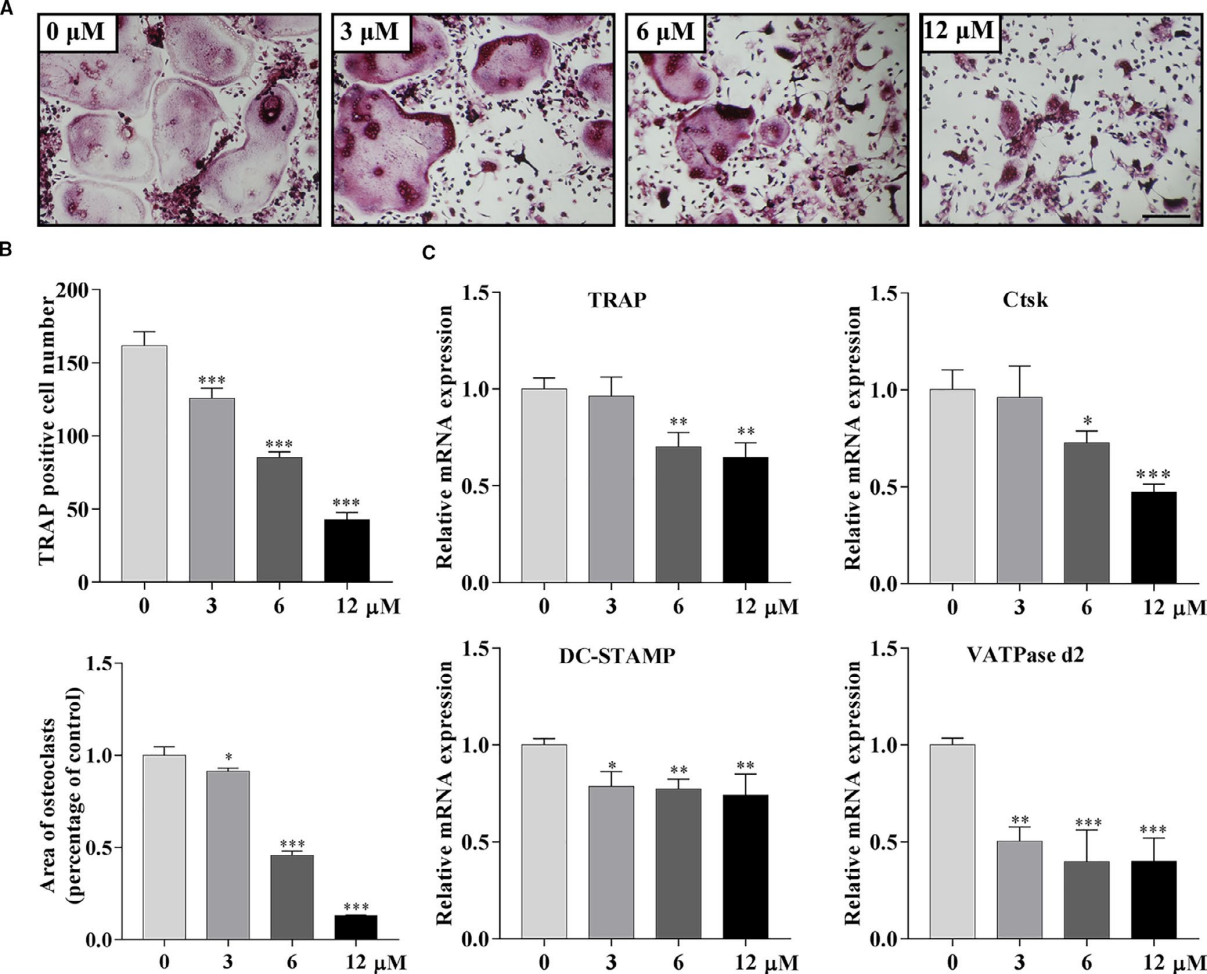
Galangin inhibits the formation of osteoclasts. (A) TRAP‐positive BMMs treated with 30 ng/mL M‐CSF, 100 ng/mL RANKL and galangin at the concentrations of 0, 1, 3, 6, 12 or 25 μmol/L for 6 days. Scale bar, 200 μm. (B) The numbers and areas of TRAP‐positive multinuclear cells were analysed using Image‐Pro Plus software. (C) Real‐time PCR quantitative analysis of TRAP, CtsK, V‐ATPase d2 and DC‐STAMP of BMMs treated with galangin at the indicated concentrations for 6 days. Data are represented as the mean ± SD *P < .05, **P < .01, ***P < .001, compared with control group

The differentiation of osteoclasts was inhibited by galangin treatment in a concentration‐dependent manner. In the control group, the TRAP‐positive cell number was shown to be 161.7 ± 9.61 per well. However, the formation of osteoclasts was inhibited following the treatment with galangin. The number of TRAP‐positive cells declined to 42.7 ± 4.93 (*P* < .001) per well after treatment with 12 μmol/L galangin. Additionally, the areas of osteoclasts dropped to 13.5 ± 0.40% (*P* < .001) in the 12 μmol/L galangin‐treated group compared with that in the control group (Figure 2A and 2B). The inhibitory effects of galangin on osteoclast differentiation were further determined by analysing the mRNA levels of down‐regulated osteoclast‐related genes. The mRNA expression of TRAP and CtsK was attenuated in a galangin concentration‐dependent manner. A similar trend was observed for V‐ATPase d2 and DC‐STAMP (Figure 2C). Therefore, these results indicate that galangin suppressed the differentiation of osteoclasts.

### 3.3 Galangin suppresses the bone resorptive activity of osteoclasts

**FIGURE 3:**
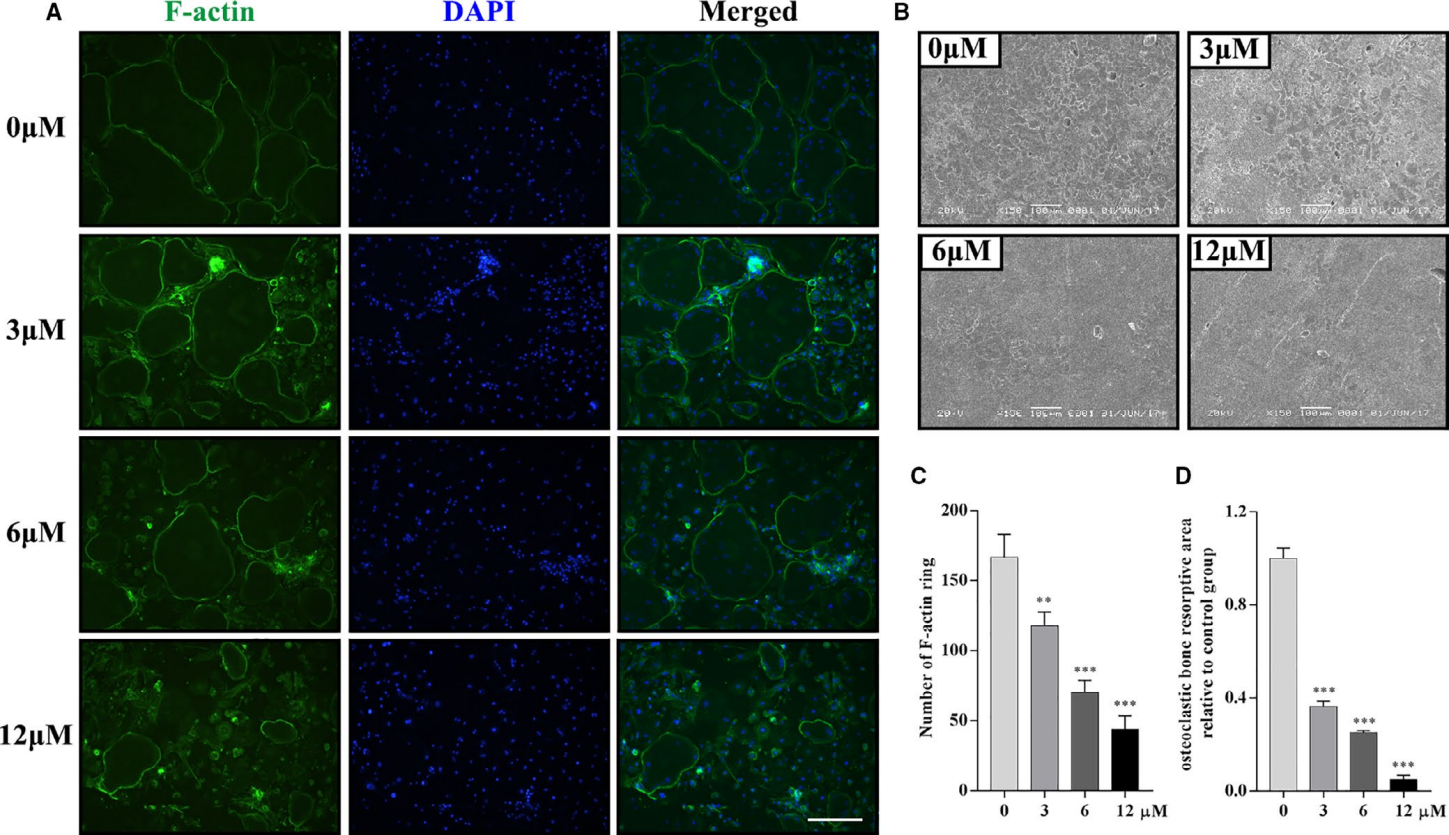
Galangin suppresses the differentiation and bone resorption of osteoclasts. (A) The F‐actin ring staining of osteoclasts treated with 30 ng/mL M‐CSF, 100 ng/mL RANKL and galangin at the concentrations of 0, 3, 6 or 12 μmol/L for 6 days. Scale bar, 200 μm. (B) BMMs were seeded on bone slices and were treated with the indicated concentrations of galangin for 10 days. The images of bone resorption pits were captured under SEM. Scale bar, 100 μm. (C‐D) The number of F‐actin rings and the areas of bone resorption were measured by Image‐Pro Plus software. Data are represented as the mean ± SD **P < .01, ***P < .001, compared with control group

Since galangin inhibited the formation of osteoclasts and the expression of osteoclast‐specific genes, its effects on in vitro osteoclastic bone resorption were examined. Galangin treatment dose‐dependently impaired the formation of mature F‐actin rings (Figure 3A). The treated osteoclasts showed smaller and fewer F‐actin rings than the control group (Figure 3C). Consistent with these results, the areas of osteoclast‐induced bone resorption pits dropped to 36.3 ± 2.31% (*P* < .001), 25.2 ± 0.85% (*P* < .001) and 5.14 ± 1.73% (*P* < .001) of that in the control group in the 3, 6 and 12 μmol/L galangin‐treated groups, respectively (Figure 3B and 3D). Collectively, our data show that galangin attenuated bone resorption and the formation of mature F‐actin rings.

### 3.4 Galangin inhibits RANKL‐induced activation of the MAPK and NF‐κB pathways

**FIGURE 4:**
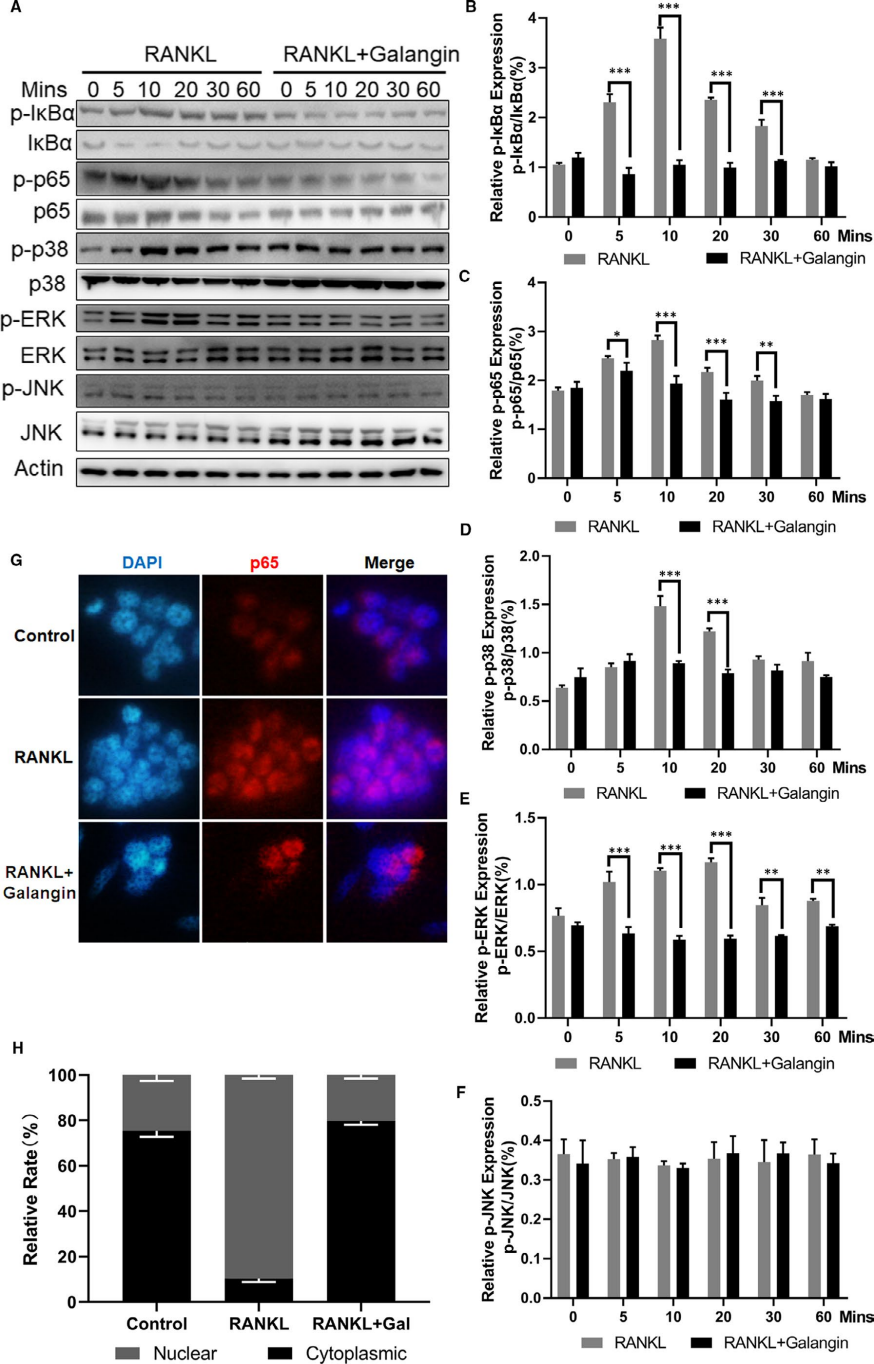
Galangin inhibits p38, ERK and NF‐κB signalling pathways. (A) BMMs were treated with 100 ng/mL RANKL and with or without 12 μmol/L galangin for the indicated time. The expression of p‐p38, p38, p‐JNK, JNK, p‐ERK, ERK and IκB‐α was measured using Western blotting. (B‐F) Quantitative analysis of indicated proteins using ImageJ software. (G) Nuclear translocation of p65 was visualized by immunofluorescence. (H) Quantification of P65 nuclear localization and cytoplasmic localization. Data are represented as the mean ± SD *P < .05, **P < .01, ***P < .001, compared with control group

**FIGURE 5:**
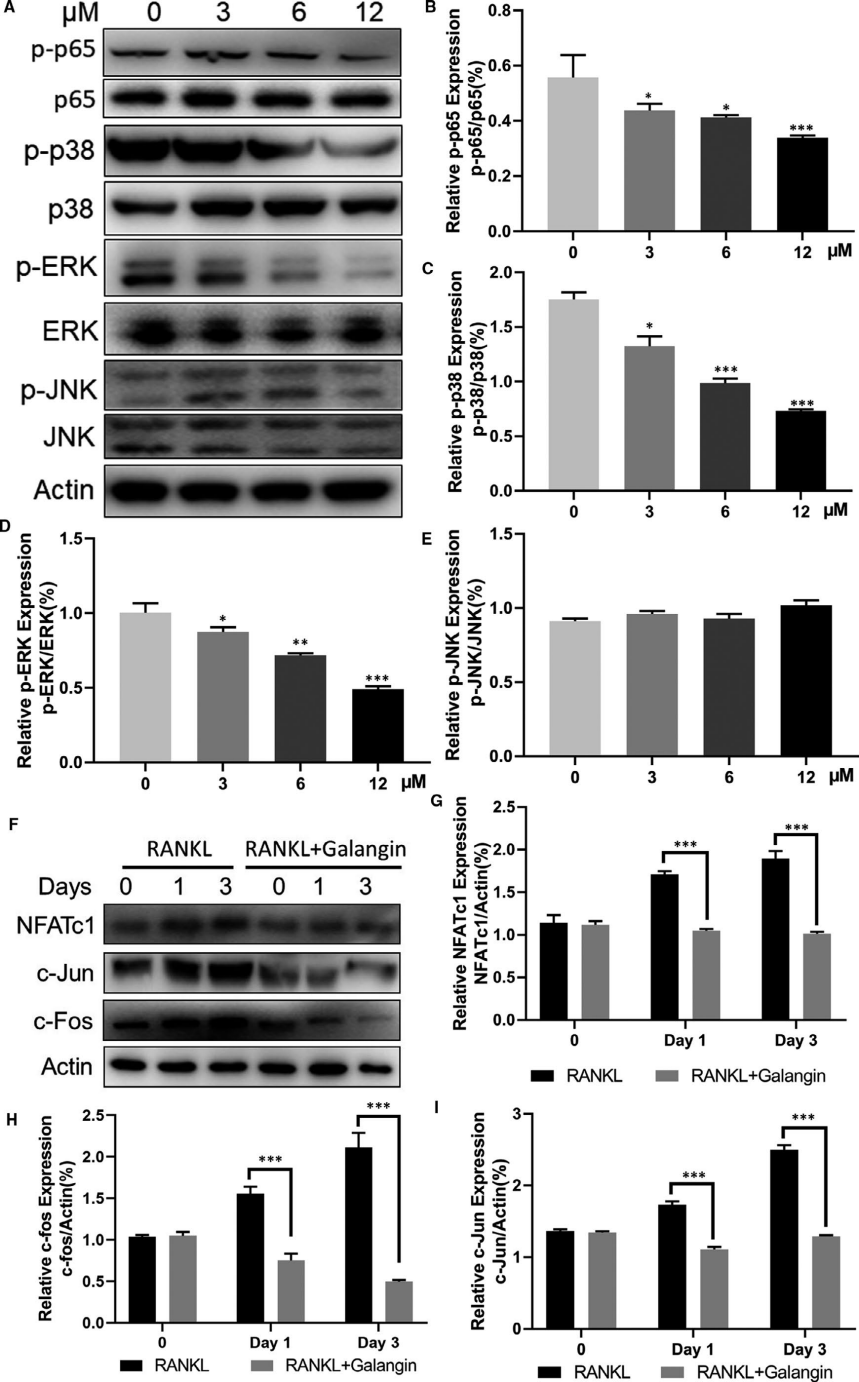
Galangin attenuates MAPK and NF‐κB signalling pathways and NFATc1 activity in a dose‐ and time‐dependent manner. (A‐E) BMMs were pre‐treated with different concentrations of galangin for 1 h and then stimulated with RANKL for 30 min. The protein expressions of p‐p65/p65, p‐p38/p38, p‐ERK/ERK, p‐JNK/JNK and actin were checked by using Western blot analysis. (F‐I) BMMs were treated with RANKL combined with or without galangin for 3 days. The images of the Western blots representing the effect of galangin on C‐Fos, C‐Jun and NFATc1 from 0, 1 and 3 days and the ratios of intensity of C‐Fos, C‐Jun and NFATc1 relative to actin were calculated using ImageJ software. Data are represented as the mean ± SD *P < .05, **P < .01, ***P < .001, compared with control group

To further elucidate the mechanism underlying galangin‐induced inhibition of suppression of osteoclast formation, we investigated the signalling pathways involved in osteoclastogenesis. Here, the galangin‐treated group showed the phosphorylation of IκBα and p65 was inhibited by galangin treatment, where the total IκB‐α level dropped rapidly after RANKL induction alone for 10 minutes and recovered at the 30 minutes time‐point (Figure 4A, B, C). Galangin markedly inhibited RANKL‐induced activation and phosphorylation of MAPK members: ERK and p38, which increased and reached their highest phosphorylation level after RANKL induction alone for 10 minutes (Figure 4A, D, E). However, the activation and phosphorylation level of JNK did not change after galangin treatment compared with that in the control group (Figure 4F). To further confirm the dose‐dependent inhibitory effect of galangin on the MAPK and NF‐κB signalling pathways, BMMs were pre‐treated with different concentrations of galangin for 1 hour and then stimulated with RANKL for 30 minutes, and the activation and phosphorylation of p65, p38 and ERK (Figure 5A, B, C, D) were decreased as galangin concentration increased except JNK (Figure 5E). To evaluate the effects of galangin on the expressions of NFATC1, C‐Jun and C‐Fos, BMMs were stimulated with RANKL without or with galangin for 0, 1 and 3 days. The expression of NFATC1, C‐Jun and C‐Fos increased in a time‐dependent manner at 0, 1 and 3 days as a result of stimulation with RANKL. However, the activation of NFATC1, C‐Jun and C‐Fos was strongly inhibited with galangin treatment (Figure 5F, G, H, I). Additionally, Immunofluorescence staining of p65 was performed with or without galangin in the induction of RANKL and M‐CSF. The results of immunofluorescence staining showed that most of the p65 was located in the cytoplasm. P65 was phosphorylated and translocated to the nucleus after induction with RANKL and M‐CSF in BMMs. However, the nuclear translocation of p65 was blocked by galangin treatment (Figure 4G, H). Therefore, the inhibitory effects of galangin on osteoclasts may involve the MAPK and NF‐κB signalling pathways.

### 3.5 Galangin prevents LPS‐induced bone loss in vivo

**FIGURE 6:**
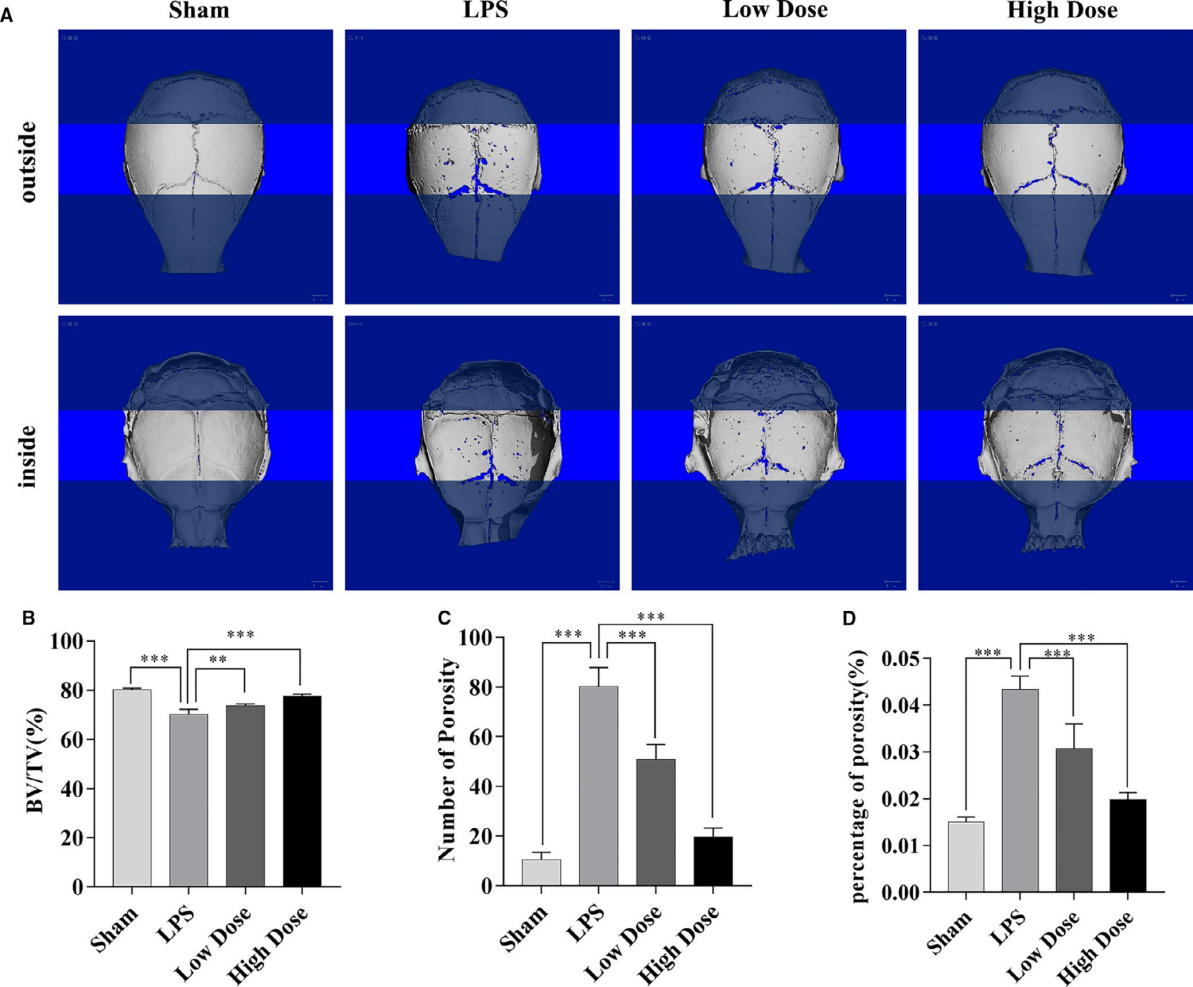
Galangin protects against LPS‐induced bone loss in vivo. (A) The fixed calvaria of mice treated with PBS, LPS and LPS + galangin (low dose or high dose) were analysed by micro‐CT, and three‐dimensional reconstructed images are presented. Scale bar, 1.0 mm. (B‐D) The percentage of bone volume to tissue volume (BV/TV), the number of porosities and the percentage of porosity were measured and analysed. Data are represented as the mean ± SD **P < .01, ***P < .001, compared with control group

**FIGURE 7:**
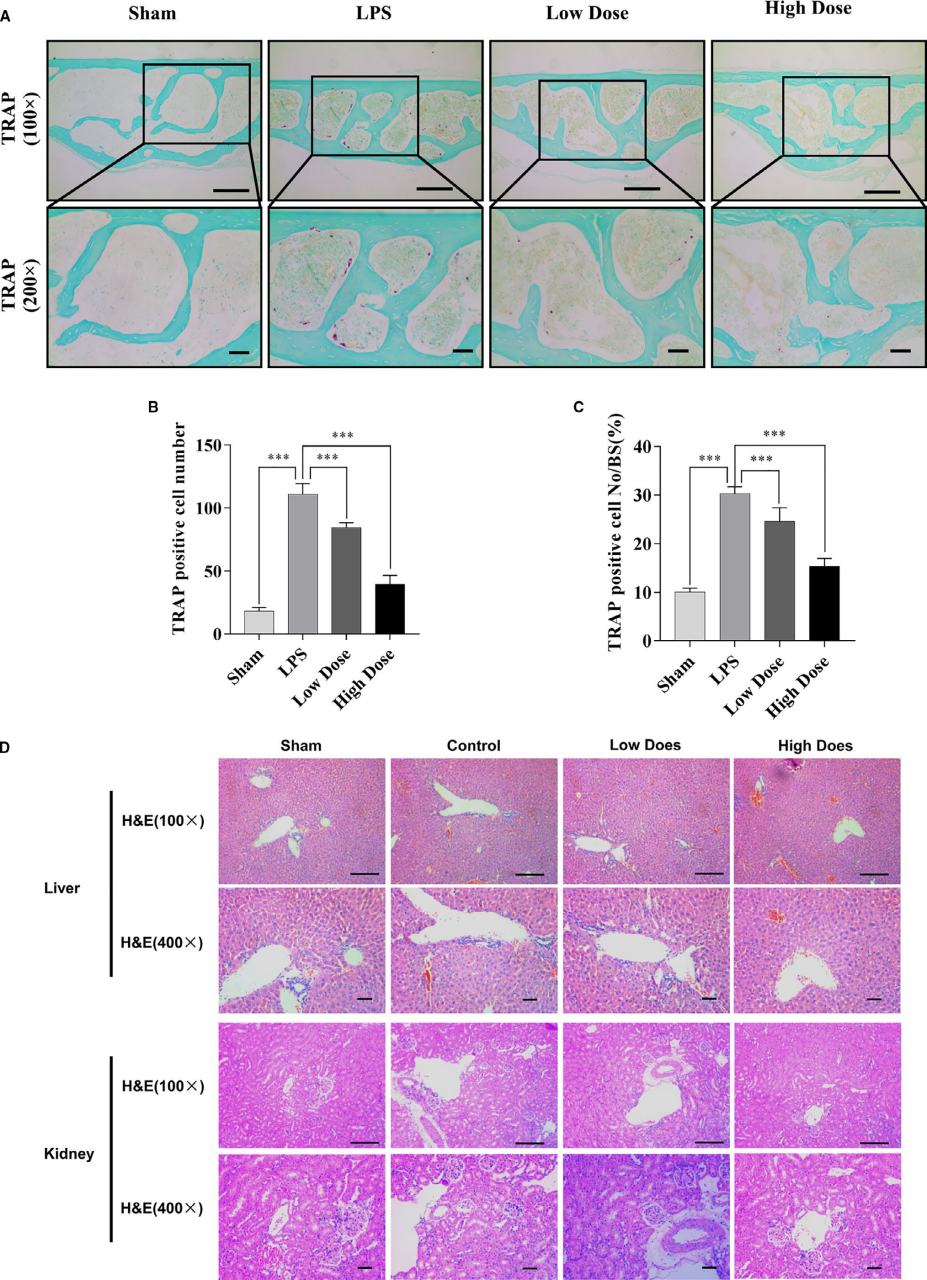
Histological analysis of the effect of galangin on LPS‐induced bone loss in vivo. (A) TRAP (magnification, 100× or 200×) staining, respectively, of calvaria sections. (B‐C) The number of TRAP‐positive cells and OcS/BS% were measured using ImageJ software. (D) H&E staining (magnification, 100× or 400×) revealed that galangin had no toxic effect on liver and kidney. Data are represented as the mean ± SD ***P < .001, compared with control group

To explore the potential protective effect of galangin in vivo, an LPS‐induced calvarial osteolytic mouse model was used. Mice were administered local subcutaneous injections of LPS to the sagittal suture of the calvarium in the absence or presence of galangin for seven days. Then, micro‐CT scanning and 3D reconstruction were conducted using the calvarial samples. The result showed that the galangin‐treated groups presented fewer calvarial osteolysis than the LPS group (Figure 6A). In the morphometric statistical analysis, BV/TV exhibited pronounced reduction of 73.7 ± 0.77% (*P* < .001) and 77.7 ± 0.68% (*P* < .001) in the low‐dose and high‐dose galangin‐treated groups, respectively, compared with that of 70.1 ± 2.23% in the LPS group (Figure 6B). A similar trend was observed for the number of porosities and percentage of porosity (Figure 6C, D). Mice administered galangin exhibited fewer TRAP‐positive multinucleated osteoclasts (Figure 7A, B, C). In addition, the pathological examination of liver and kidney has been done to explore the side‐effects of galangin in our mice models. Interestingly, H&E staining revealed that galangin had no toxic effect on liver and kidney (Figure 7D). Collectively, our data suggest that galangin protects against LPS‐induced bone loss in vivo.

## 4 DISCUSSION

In our study, we identify and characterize the effects of galangin, a well‐known component of traditional Chinese medicine, on the differentiation and the function of osteoclasts. We found that galangin inhibited multinucleation, the formation of F‐actin rings and the bone resorptive activity of osteoclasts, which was confirmed by the reduced expression of osteoclast‐specific genes, including TRAP, Ctsk, DC‐STAMP and V‐ATPase d2. Furthermore, galangin suppressed RANKL‐induced ERK‐MAPK, p38‐MAPK and *NF‐κB* signalling pathways by attenuating phosphor‐ERK, phosphor‐p38 and phosphor‐p65, and stabilizing IκB‐α expression in BMMs. Considering the above results, we speculate that galangin directly inhibits osteoclastogenesis via NF‐κB and MAPK signalling pathways.

After receiving signals transduced by ligand activation of RANK, osteoclasts undergo a series of internal structural changes to prepare for bone resorption. The actin cytoskeleton is rearranged and the ruffled border, a notable feature of polarized osteoclasts, is formed.^5^ Meanwhile, secretion of lytic enzymes, such as TRAP and pro‐CTSK, is continually exported into the resorption pit, along with hydrogen ions. Here, we demonstrated that multinucleation and the formation of the F‐actin rings, as well as the bone resorptive activity, were inhibited in a concentration‐dependent manner after treatment with galangin, indicating galangin directly inhibits the differentiation and function of osteoclasts. Interestingly, galangin did not enhance osteoclast differentiation within its non‐toxic dose, while quercitrin and taxifolin have bone‐forming activity by stimulating osteoblast differentiation.^12^ This discrepancy may be attributed to the slight structural differences between these compounds.

Temporal expression of osteoclast phenotype markers during osteoclast differentiation is characterized by an increase in TRAP after RANKL induction, an increase in DC‐STAMP and V‐ATPase d2 during cell‐cell fusion, and an increase in Ctsk during bone formation. TRAP is expressed in the early stage as a typical marker of osteoclast differentiation and is capable of catalysing hydrolysis of phosphate esters and anhydrides under acidic conditions ^22^ and forming reactive oxygen species.^23^ Ctsk, a proteinase from the ruffled border of mature osteoclasts, dissolves the inorganic and organic components of bone in cooperation with hydrogen ions. Previous research has shown that Ctsk‐deficient osteoclasts fail to degrade collagen, elastin and gelatin.^24^ DC‐STAMP and V‐ATPase d2 participate in the process of osteoclast cell‐cell fusion, which is an indispensable event for efficient bone resorption.^25^ Thus, we selected those gene markers to reflect the effect of galangin on osteoclasts. We found that the expression of TRAP, Ctsk, DC‐STAMP and V‐ATPase d2 was down‐regulated by galangin in a concentration‐dependent manner, which further proves that galangin may directly reduce the formation and the bone resorptive activity of osteoclasts, in accordance with our TRAP staining results.

The interaction of RANKL with RANK is an essential step in the initiation of osteoclastogenesis. With the beginning of RANKL‐RANK binding, a variety of intracellular signalling pathways involving *NF‐κB*, MAPKs and PI3K/Akt are activated in the osteoclast precursors. *NF‐κB* and IκB make up a complex, which resides in the cytoplasm in a normal state. NF‐κB dimers are activated by IKK‐mediated phosphorylation of IκB, which triggers proteasomal IκB degradation. This enables the active NF‐κB transcription factor subunits to translocate to the nucleus and induce target gene expression. p38‐MAPK signalling functions in the early stage of osteoclast differentiation as it positively regulates the expression of MIIF and TRAP, while the application of a specific inhibitor of ERK has been shown to prevent osteoclast formation. Meanwhile, the overexpression of JNK enhances RANKL‐induced osteoclastogenesis.^26^ Further, different flavonoids may work through different mechanisms due to their structural differences. Licochalcone A and naringin inhibited osteoclast through inhibition of *NF‐κB* and ERK‐MAPK, rather than p38‐MAPK or JNK‐MAPK.^27^ Herbacetin inhibited osteoclast through inhibition of *NF‐κB* and JNK‐MAPK rather than p38‐MAPK or ERK‐MAPK.^20^ In our study, we demonstrated that galangin significantly inhibited p38, ERK and NF‐κB signalling pathways by inhibiting the phosphorylation of proteins in BMMs, with little effect in JNK signalling. These data, consistent with our q‐PCR results, suggest that RANKL‐mediated osteoclast differentiation is suppressed, and p38‐MAPK, ERK‐MAPK and *NF‐κB* signalling pathways may serve as potential targets in osteoclastogenesis inhibition when treated with galangin (Figure 2). NFATc1 serves as a key central transcription factor in osteoclastogenesis. NFATc1 induces the marker gene expression of mature OCs, such as TRAP and CTSK. In this study, we demonstrated that galangin inhibits RANKL‐induced activation of NFATc1 in a time‐dependent manner.

LPS, a Gram‐negative endotoxin, induces bone resorption via recruitment of macrophages, lymphocytes, gingival and fibroblasts. After the recruitment of LPS cells, PGE2, IL‐6 and TNF‐α are released into the microenvironment, which in turn activates osteoclasts. Thus, we relied on the LPS‐induced calvarial osteolytic mouse model to assess galangin's protective effect against bone loss. Our data demonstrated that administration of galangin significantly reduced bone resorption and the number of TRAP‐positive osteoclasts, which suggests that the inhibition of LPS‐induced bone loss may be attributed to the increased activation of osteoclasts. In our previous study, we found that galangin could suppress osteosarcoma cells by inhibiting their proliferation and invasion and accelerating their apoptosis, and the mechanism may be associated with the inhibition of PI3K and its downstream signalling pathway.^28^ Daniel Branstetter reported that the targeting of the bone microenvironment by inhibition of osteoclastogenesis prevents tumour‐induced osteolysis and subsequent skeletal complications.^29^ We speculate that galangin has dual functions in the inhibition of osteosarcoma cells, as well as osteoclasts in the process of inhibiting invasion and metastasis of osteosarcoma. Those results indicate galangin may inhibit lung metastasis of osteosarcoma by inhibiting the function of osteoclasts, which needs to be further explored. In addition, galangin has no obvious effect on the proliferation of MC3T3‐E1 osteoblast cells in the range of 0‐25μM, but it can promote the differentiation and bone mineralization of MC3T3‐E1 osteoblast cells in the range of 0‐12μM through experiments of MTT assay, ALP staining and Alizarin Red Staining (Figures S1, S2).

There are several limitations in this study. First, a positive control group, such as a bisphosphonates‐treated group, was not included. Secondly, the explanation for the influence on p38 and ERK rather than JNK signalling by galangin remains unclear, and the target molecule that galangin directly affects needs to be further explored. Additionally, the LPS‐inducted mouse calvarial model is not identical to the physio‐pathologic processes of osteolysis in human patients. Further experiments need to be conducted on large animals or humans to confirm the efficacy of galangin.

In conclusion, we found a novel pharmacological role of galangin, that is galangin could effectively suppress the development and progress of osteoporosis through suppression of osteoclastogenesis via inhibition of p38, ERK and *NF‐κB* signalling pathways. We believe that galangin merits further studies and galangin has presented as a promising agent against osteoporosis.

## CONFLICT OF INTEREST

The authors confirm that there are no conflicts of interest.

## AUTHOR CONTRIBUTION

**Xiucheng Li:** Data curation (equal); Investigation (equal); Writing–original draft (equal); Writing–review & editing (equal). **Jiawei Jiang:** Data curation (equal); Methodology (equal). **Zhifan Yang:** Data curation (equal); Methodology (equal). **Songtao Jin:** Investigation (equal); Software (equal); Visualization (equal). **Xuanyuan Lu:** Investigation (equal); Software (equal); Visualization (equal). **Yu Qian:** Project administration (equal); Supervision (equal); Writing–review & editing (equal).

## Supporting information

Fig S1

Fig S2

## Data Availability

All data generated or analysed during this study are included in this article.
