# Supplementary figures and images for: Galangin suppresses RANKL‐induced osteoclastogenesis via inhibiting MAPK and NF‐κB signalling pathways
Source: J Cell Mol Med. 2021 May 3;25(11):4988–5000. doi: 10.1111/jcmm.16430 (PMC8178255; doi:10.1111/jcmm.16430)

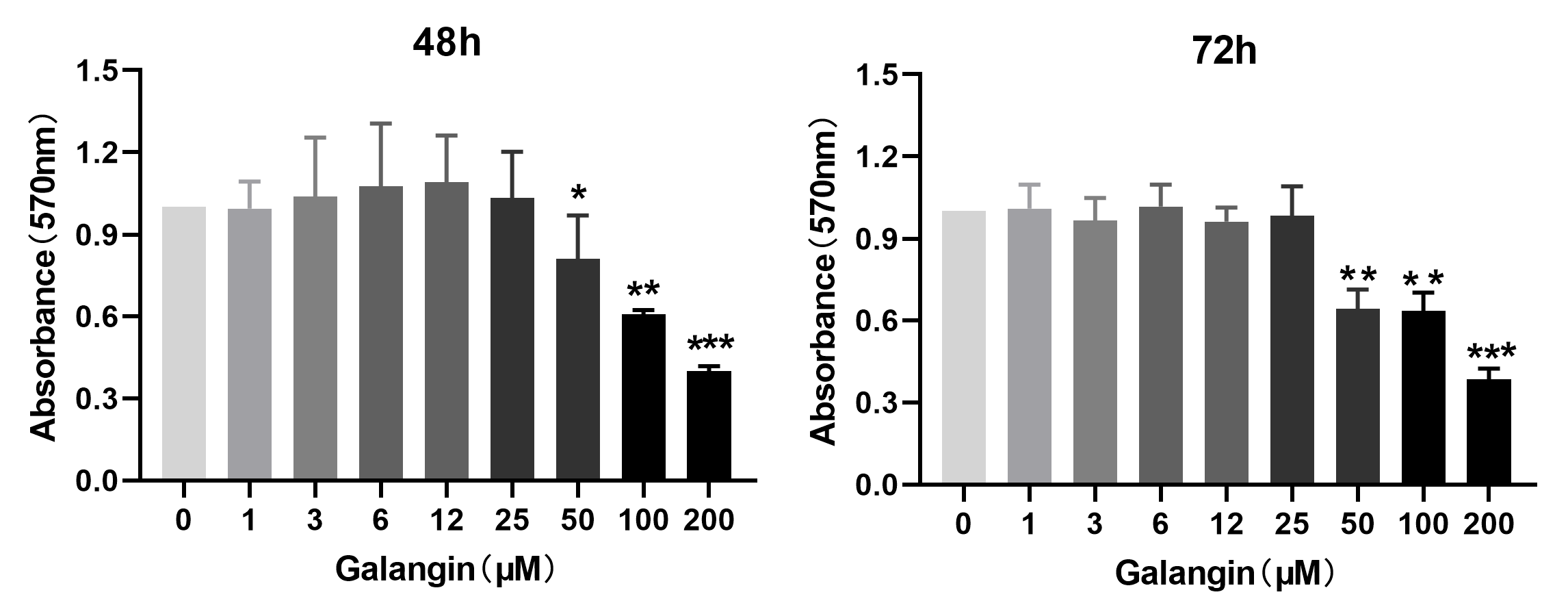

Supplement: Supplementary file 1 — Fig S1 [file JCMM-25-4988-s002.tif]

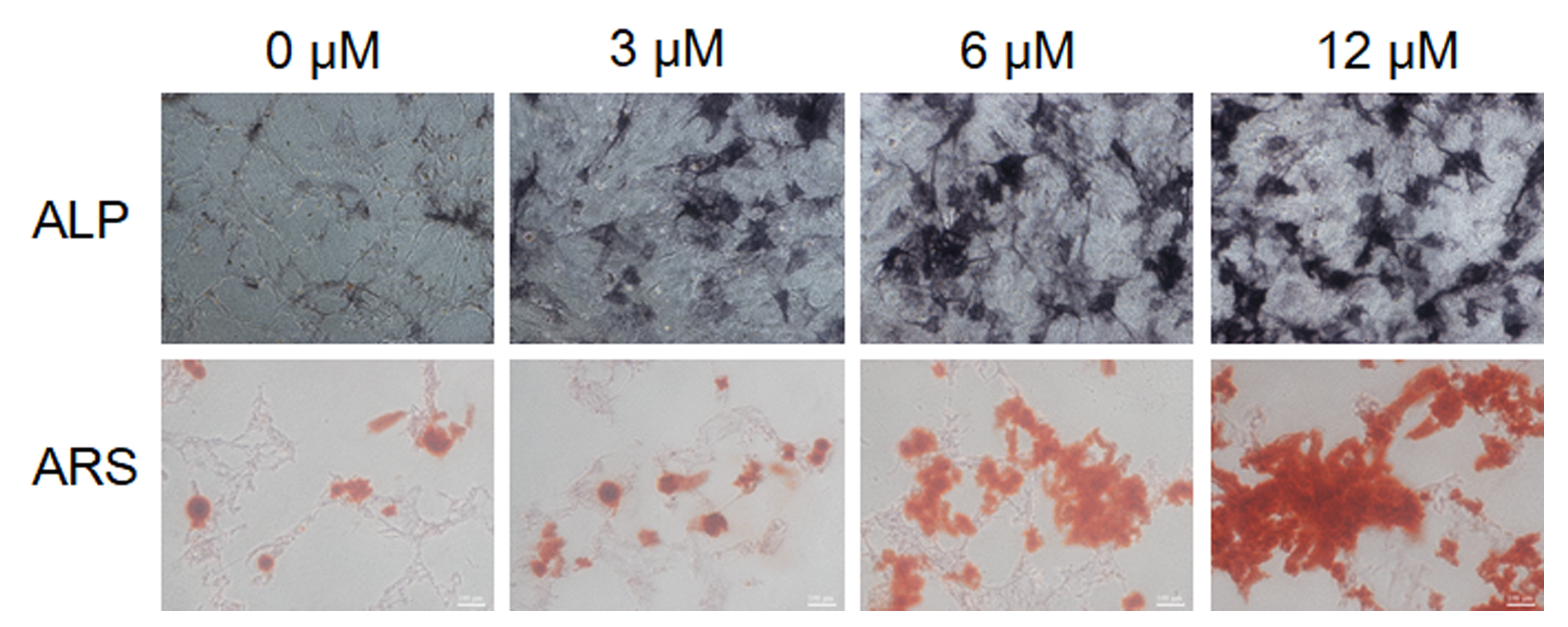

Supplement: Supplementary file 2 — Fig S2 [file JCMM-25-4988-s001.tif]
